# Supplementary material for: The Impacts of Prenatal Mental Health Issues on Birth Outcomes during the COVID-19 Pandemic: A Scoping Review
Source: Int J Environ Res Public Health. 2022 Jun 23;19(13):7670. doi: 10.3390/ijerph19137670 (PMC9265353; doi:10.3390/ijerph19137670)
Supplement: Supplementary file 1 [file ijerph-19-07670-s001.zip › ijerph-1732178-supplementary.pdf]

Supplementary Materials

Table S1. Detailed search strings

Ovid MEDLINE(R) ALL <1946 to July 26, 2021>

#	Search Statement	Results
1	exp Coronavirus/	85818
2	exp Coronavirus Infections/	104836
3	(coronavirus* or corona virus* or OC43 or NL63 or 229E or HKU1 or HCoV* or ncov* or covid* or sars-cov* or sarscov* or Sars-coronavirus* or Severe Acute Respiratory Syndrome Coronavirus*).mp.	176694
4	(or/1-3) and 20200601:20301231.(ep).	115200
5	4 not (SARS or SARS-CoV or MERS or MERS-CoV or Middle East respiratory syndrome or camel* or dromedar* or equine or coronary or coronal or covidence* or covidien or influenza virus or HIV or bovine or calves or TGEV or feline or porcine or BCoV or PED or PEDV or PDCoV or FIPV or FCoV or SADS-CoV or canine or CCov or zoonotic or avian influenza or H1N1 or H5N1 or H5N6 or IBV or murine corona*).mp.	46230
6	((pneumonia or covid* or coronavirus* or corona virus* or ncov* or 2019-ncov or sars*).mp. or exp pneumonia/) and Wuhan.mp.	5588
7	(2019-ncov or ncov19 or ncov-19 or 2019-novel CoV or sars-cov2 or sars-cov-2 or sarscov2 or sarscov-2 or Sars-coronavirus2 or Sars-coronavirus-2 or SARS-like coronavirus* or coronavirus-19 or covid19 or covid-19 or covid 2019 or ((novel or new or nouveau) adj2 (CoV on nCoV or covid or coronavirus* or corona virus or Pandemi*2)) or ((covid or covid19 or covid-19) and pandemic*2) or (coronavirus* and pneumonia)).mp.	161412
8	COVID-19.rx,px,ox. or severe acute respiratory syndrome coronavirus 2.os.	4645
9	Covid-19/	94250
10	(variant* adj2 (India* or "South Africa*" or UK or English or Brazil* or alpha or beta or delta or gamma or kappa or lambda or "P.1" or "C.37")).mp.	4449
11	("B.1.1.7" or "B.1.351" or "B.1.617.1" or "B.1.617.2").mp.	530
12	or/5-11	167457
13	12 and 20191101:20301231.(dt).	161454
14	exp Pregnancy Complications/	442678
15	exp Pregnancy Outcome/	77812
16	exp Obstetric Labor Complications/	72047
17	exp Obstetric Labor, Premature/	28601
18	exp Fetal Death/	29935
19	"congenital, hereditary, and neonatal diseases and abnormalities"/	890
20	("adverse birth outcome*" or stillbirth* or "low birthweight*" or "low birth weigh" or "small for gestational age" or SGA or macrosomia or "neonatal death" or "congenital anomal*").mp.	66064
21	("spontaneous abortion*" or "postpartum hemorhage" or "post partum hemorhage").mp.	10894
22	((labor or labour or deliver* or childbirth* or birth*) adj3 (prematur* or pre-mature* or preterm or pre-term or early or difficult*)).mp.	70898
23	or/14-22	527852
24	exp Mental Health/	45210
25	exp Psychology/	68604
26	exp Psychiatry/	106292
27	Anxiety/	89498
28	Depression/	130306
29	(mental* adj3 (ill* or well* or disorder* or disease* or health* or unwell*)).mp.	403543
30	anxiety disorders/ or agoraphobia/ or anxiety, separation/ or neurocirculatory asthenia/ or neurotic disorders/ or obsessive-compulsive disorder/ or hoarding disorder/ or panic disorder/ or phobic disorders/ or phobia, social/	82771
31	(((anxiety or depression or depressive or hypervigilance or agoraphobia or neuroses or neurosis or neurotic or paranoi* or catastrophiz* or catastorphis*or) and obsessive-compulsive) or panic or phobia or stress or ptsd or psycho* or "internalising symptom*" or "internalizing symptom*" or wellness or "seasonal affective disorder*" or psychiatr*).mp.	2890504
32	(dysthymic disorder* or "use disorder" or "psychotropic drug*" or "brain disease*" or "neurotransmitter agent*" or cognitive or "social problems" or "alcohol abuse*" or "drug abuse*").mp.	594133
33	("seasonal affective disorder*" or "internali?ing symptoms" or "common mental disorders" or cmd or cmds).ti,ab.	8063
34	or/24-33	3387821
35	13 and 23 and 34	231

Embase <1974 to 2021 July 26>

#	Search Statement	Results
1	((exp Coronavirus/ or exp Coronavirus Infections/ or (coronavirus* or corona virus* or OC43 or NL63 or 229E or HKU1 or HCoV* or ncov* or covid* or sars-cov* or sarscov* or Sars-coronavirus* or Severe Acute Respiratory Syndrome Coronavirus* or D614G).mp.) not (SARS or SARS-CoV or MERS or MERS-CoV or Middle East respiratory syndrome or camel* or dromedar* or equine or coronary or coronal or covidence* or covidien or influenza virus or HIV or bovine or calves or TGEV or feline or porcine or BCoV or PED or PEDV or PDCoV or FIPV or FCoV or SADS-CoV or canine or CCov or zoonotic or avian influenza or H1N1 or H5N1 or H5N6 or IBV or murine corona*).mp.) or coronavirus disease 2019/ or (((pneumonia or covid* or coronavirus* or corona virus* or ncov* or 2019-ncov or sars*).mp. or exp pneumonia/) and Wuhan.mp.) or ("coronavirus disease 2019" or "2019 ncov" or ncov19 or ncov-19 or "2019 novel CoV" or severe acute respiratory syndrome coronavirus 2 or sars-cov2 or sars-cov-2 or sarscov2 or sarscov-2 or Sars-coronavirus2 or Sars-coronavirus-2 or SARS-like coronavirus* or coronavirus-19 or covid19 or covid-19 or "covid 2019" or "B.1.1.7" or "B.1.351" or "B.1.617.1" or "B.1.617.2" or ((cov* or corona* or SARS) and (variant* adj2 (India* or "South Africa*" or UK or English or Brazil* or alpha or beta or delta or gamma or kappa or lambda or "P.1" or "C.37"))) or ((novel or new or nouveau) adj2 (CoV or nCoV or coronavirus* or corona virus))).mp.	175153
2	"parameters concerning the fetus, newborn and pregnancy"/ or apgar score/ or birth weight/ or fetus mortality/ or fetus outcome/ or fetus risk/ or fetus weight/ or gestational age/ or live birth/ or exp perinatal morbidity/ or perinatal mortality/ or pregnancy outcome/ or prenatal mortality/ or exp fetus death/	316934
3	exp labor complication/ or exp pregnancy complication/	308606
4	birth/ or birth weight/ or caesarean birth/ or "labor (childbirth)"/ or natural childbirth/ or premature birth/ or birth injuries/ or birth trauma/	244903
5	(exp congenital disorder/ and (neonat* or newborn*).mp.) or newborn disease/ or dysmaturity/ or immaturity/ or large for gestational age/ or low birth weight/ or neonatal alloimmune thrombocytopenia/ or neonatal hemorrhage/ or neonatal hyperbilirubinemia/ or neonatal pneumonia/ or neonatal respiratory distress syndrome/ or neonatal stress/ or neonatal thrombocytopenia/ or newborn anemia/ or newborn apnea/ or newborn diabetes mellitus/ or newborn hemolytic disease/ or newborn hepatitis/ or newborn hypoxia/ or newborn infection/ or newborn jaundice/ or newborn ophthalmia/ or newborn sepsis/ or newborn tetanus/ or perinatal asphyxia/ or perinatal stress/ or prematurity/ or retrolental fibroplasia/ or "transient tachypnea of the newborn"/ [mp=title, abstract, heading word, drug trade name, original title, device manufacturer, drug manufacturer, device trade name, keyword, floating subheading word, candidate term word]	332734
6	("adverse birth outcome*" or stillbirth* or "low birthweight*" or "low birth weigh" or "small for gestational age" or SGA or macrosomia or "neonatal death" or "congenital anomal*").mp.	93650
7	(((labor or labour or deliver* or childbirth* or birth*) adj3 (prematur* or pre-mature* or preterm or pre-term or early or difficult*)) or "spontaneous abortion*").mp.	142238
8	or/2-7	806020
9	exp mental health/	178312
10	exp psychology/	367416
11	exp mental disease/	2317236
12	((mental* or psychiatric*) adj3 (ill* or well* or disorder* or disease* or health* or unwell*)).mp.	609171
13	(((anxiety or depression or depressive or hypervigilance or agoraphobia or neuroses or neurosis or neurotic or paranoi* or catastrophiz* or catastorphis*or and obsessive-compulsive or panic or phobia or stress or ptsd or psycho* or "internalising symptom*" or "internalizing symptom*" or wellness or "seasonal affective disorder*" or psychiatr*).mp.	3137713
14	(dysthymic disorder* or "use disorder" or "psychotropic drug*" or "brain disease*" or "neurotransmitter agent*" or cognitive or "social problems" or "alcohol abuse*" or "drug abuse*").mp.	846971
15	neurocirculatory asthenia.mp.	160
16	major depression/ or dysthymic disorder/ or atypical depression/ or internalizing symptoms/ or seasonal affective disorder/ or mental health/ or primary mental health prevention/	227428
17	(depression or ("common mental disorders" or cmd or cmds)).mp.	731977
18	or/9-17	4933377
19	1 and 8 and 18	425
20	limit 19 to dc=20190601-20210726	421

APA PsycInfo <1806 to July Week 3 2021>

#	Search Statement	Results
1	((exp Coronavirus/ or (coronavirus* or corona virus* or OC43 or NL63 or 229E or HKU1 or HCoV* or ncov* or covid* or sars-cov* or sarscov* or Sars-coronavirus* or Severe Acute Respiratory Syndrome Coronavirus* or D614G).mp.) not (SARS or SARS-CoV or MERS or MERS-CoV or Middle East respiratory syndrome or camel* or dromedar* or equine or coronary or coronal or covidence* or covidien or influenza virus or HIV or bovine or calves or TGEV or feline or porcine or BCoV or PED or PEDV or PDCoV or FIPV or FCoV or SADS-CoV or canine or CCov or zoonotic or avian influenza or H1N1 or H5N1 or H5N6 or IBV or murine corona*).mp.) or Covid-19/ or (((pneumonia or covid* or coronavirus* or corona virus* or ncov* or 2019-ncov or sars*).mp. or exp pneumonia/) and Wuhan.mp.) or ("coronavirus disease 2019" or 2019-ncov or ncov19 or ncov-19 or 2019-novel CoV or severe acute respiratory syndrome coronavirus 2 or sars-cov2 or sars-cov-2 or sarscov2 or sarscov-2 or Sars-coronavirus2 or Sars-coronavirus-2 or SARS-like coronavirus* or coronavirus-19 or covid19 or covid-19 or covid 2019 or "B.1.1.7" or "B.1.351" or "B.1.617.1" or "B.1.617.2" or (variant* adj2 ("South Africa*" or UK or English or Brazil* or alpha or beta or delta or gamma or kappa or lambda or "P.1" or "C.37")) or ("B.1.1.7" or "B.1.351" or "B.1.617.1" or "B.1.617.2") or ((novel or new or nouveau) adj2 (CoV or nCoV or coronavirus* or corona virus))).mp.	7625
2	obstetrical complications/ or exp birth/ or birth injuries/ or caesarean birth/ or premature birth/	17831
3	pregnancy outcomes/ or exp birth/ or induced abortion/ or exp obstetrical complications/ or spontaneous abortion/ or birth injuries/ or birth weight/	22232
4	Stillbirths/	0
5	exp Congenital Disorders/	8202
6	("adverse birth outcome*" or stillbirth* or "low birthweight*" or "low birth weigh" or "small for gestational age" or SGA or macrosomia or "neonatal death" or "congenital anomal*").mp.	4037
7	(((labor or labour or deliver* or childbirth* or birth*) adj3 (prematur* or pre-mature* or preterm or pre-term or early or difficult*)) or "spontaneous abortion*").mp.	11298
8	or/2-7	35212
9	1 and 8	25
10	limit 9 to up=20190601-20211231	24

CINAHL Plus with Full Text
Limiters/Expanders
Search Modes - Find all my search terms

#	Query	Results
S1	(((MH "Coronavirus+") OR (MH "Coronavirus Infections+") or (coronavirus* or corona virus* or OC43 or NL63 or 229E or HKU1 or HCoV* or ncov* or covid* or sars-cov* or sarscov* or Sars-coronavirus* or Severe Acute Respiratory Syndrome Coronavirus*)) NOT ((SARS or SARS-CoV or MERS or MERS-CoV or Middle East respiratory syndrome or camel* or dromedar* or equine or coronary or coronal or covidence* or covidien or influenza virus or HIV or bovine or calves or TGEV or feline or porcine or BCoV or PED or PEDV or PDCoV or FIPV or FCoV or SADS-CoV or canine or CCov or zoonotic or avian influenza or H1N1 or H5N1 or H5N6 or IBV or murine corona*)) or (MH "COVID-19") OR (MH "COVID-19 Pandemic") OR (MH "SARS-CoV-2") or(covid or 2019-ncov or ncov19 or ncov-19 or 2019-novel CoV or sars-cov2 or sars-cov-2 or sarscov2 or sarscov-2 or Sars-coronavirus2 or Sars-coronavirus-2 or SARS-like coronavirus* or coronavirus-19 or ((novel or new or nouveau) N2 (CoV or nCoV or coronavirus* or “corona virus” or Pandemi*)) or (variant* N2 (India* or "South Africa*" or UK or English or Brazil* or alpha or beta or delta or gamma or kappa or lambda or "P.1" or "C.37")) or ("B.1.1.7" or "B.1.351" or "B.1.617.1" or "B.1.617.2")) and EM 20190601-20301231	61,878
S2	(MH "Pregnancy Outcomes")	25,494
S3	(MH "Pregnancy Complications+")	100,854
S4	(MH "Perinatal Death")	8,709
S5	(MH "Infant, Low Birth Weight+")	15,192
S6	(MH "Infant, Large for Gestational Age") OR (MH "Infant, Postmature") OR (MH "Infant, Premature")	24,902
S7	"adverse birth outcome*" or stillbirth* or "low birthweight*" or "low birth weigh" or "small for gestational age" or SGA or macrosomia or "neonatal death" or "congenital anomal*	25,026
S8	(((labor or labour or deliver* or childbirth* or birth*) N3 (prematur* or pre-mature* or preterm or pre-term or early or difficult*)) or "spontaneous abortion*")	31,274
S9	stillbirth* or "still birth*" or stillborn or "still born" or "intrauterine death" or "perinatal death" or "fetus death" or "fetal demise" or "fetal death"	12,791
S10	S2 OR S3 OR S4 OR S5 OR S6 OR S7 OR S8 OR S9	163,731
S11	(MH "Mental Health") OR (MH "Behavioral and Mental Disorders+")	897,469
S12	(MH "Psychology, Clinical+")	1,037
S13	((mental* or psychiatric*) N3 (ill* or well* or disorder* or disease* or health* or unwell*))	229,030
S14	(anxiety or depression or depressive or hypervigilance or agoraphobia or neuroses or neurosis or neurotic or paranoi* or catastrophiz* or catastorphis*or and obsessive-compulsive or panic or phobia or stress or ptsd or psycho* or "internalising symptom*" or "internalizing symptom*" or wellness or "seasonal affective disorder*" or psychiatr*)	1,135,565
S15	("dysthymic disorder*" or "use disorder" or "psychotropic drug*" or "brain disease*" or "neurotransmitter agent*" or cognitive or "social problems" or "alcohol abuse*" or "drug abuse*")	200,728
S16	(S11 OR S12 OR S13 OR S14 OR S15)	1,615,363
S17	S1 AND S10 AND S16	105

SCOPUS Searched July 27, 2021 Results n=95

(((TITLE-ABS-KEY ((coronavirus* OR "corona virus*" OR oc43 OR nl63 OR 229e OR hku1 OR hcov* OR ncov* OR covid* OR "sars-cov*" OR sarscov* OR "Sars-coronavirus*" OR "Severe Acute Respiratory Syndrome Coronavirus*" OR d614g))) AND NOT ((TITLE-ABS-KEY ((sars OR sars-cov OR mers OR mers-cov OR "Middle East respiratory syndrome or camel*" OR dromedar* OR equine OR coronary OR coronal OR covidence* OR covidien OR influenza AND virus OR hiv OR bovine OR calves OR tgev OR feline OR porcine OR bcov))) OR (TITLE-ABS-KEY ((ped OR pedv OR pdcov OR fipv OR fcov OR sads-cov OR canine OR ccov OR zoonotic OR "avian influenza" OR h1n1 OR h5n1 OR h5n6 OR ibv OR murine AND corona*))))) OR (TITLE-ABS-KEY ((pneumonia OR covid* OR coronavirus* OR corona AND virus* OR ncov* OR 2019-ncov OR sars*) AND wuhan) OR ((2019-ncov OR ncov19 OR ncov-19 OR 2019-novel AND cov OR sars-cov2 OR sars-cov-2 OR sarscov2 OR sarscov-2 OR sars-coronavirus2 OR sars-coronavirus-2 OR "SARS-like coronavirus*" OR coronavirus-19 OR covid19 OR covid-19 OR "covid 2019" OR "B.1.1.7" OR "B.1.351" OR "B.1.617.1" OR "B.1.617.2" OR (variant* W/2 (india* OR "South Africa*" OR uk OR english OR brazil* OR alpha OR beta OR delta OR gamma OR kappa OR lambda OR "P.1" OR "C.37")) OR ((covid OR covid19 OR covid-19) AND pandemic*) OR (coronavirus* AND pneumonia)))) OR (TITLE ((novel OR new OR nouveau) AND (cov OR ncov OR covid OR coronavirus* OR corona AND virus OR pandemi*))) OR (ABS ((novel OR new OR nouveau) AND (cov OR ncov OR covid OR coronavirus* OR corona AND virus OR pandemi*))) OR (KEY ((novel OR new OR nouveau) AND (cov OR ncov OR covid OR coronavirus* OR corona AND virus OR pandemi*))) AND ORIG-LOAD-DATE > 20190630) AND (TITLE-ABS-KEY ("birth outcome*" OR "birth complication*" OR stillbirth* OR "fetal death*" OR "low birthweight*" OR "low birth weigh" OR "small for gestational age" OR sga OR macrosomia OR "neonatal death" OR "congenital anomal*" OR "pregnancy outcome*" OR "obstetrical outcome*" OR "obstetrical complication*" OR "labor complication*" OR "spontaneous abortion*") OR TITLE-ABS-KEY ((labor OR labour OR deliver* OR childbirth* OR birth*) W/3 (prematur* OR pre-mature* OR preterm OR pre-term OR early OR difficult*))) AND (TITLE-ABS-KEY ((mental* W/3 (ill* OR well* OR disorder* OR disease* OR health* OR unwell*))) OR TITLE-ABS-KEY ((anxiety OR depression OR depressive OR hypervigilance OR agoraphobia OR neuroses OR neurosis OR neurotic OR paranoi* OR catastrophiz* OR catastorphis*or AND obsessive-compulsive OR panic OR phobia OR stress OR ptsd OR psycho* OR "internalising symptom*" OR "internalizing symptom*" OR wellness OR "seasonal affective disorder*" OR psychiatr*)) OR TITLE-ABS-KEY ("use disorder" OR "psychotropic drug*" OR "brain disease*" OR "neurotransmitter agent*" OR cognitive OR "social problems" OR "alcohol abuse*" OR "drug abuse*"))

Cochrane Library (CDSR and Central Register of Controlled Trials)
Searched July 27, 2021

ID	Search	Hits
#1	MeSH descriptor: [COVID-19] explode all trees	467
#2	(covid19 or "covid 19" or "coronavirus 2019" or "corona virus 2019" or "sars cov2"):ti,ab,kw	5991
#3	variant* near (india* or "south african" or England or UK or Brazil or alpha or beta or delta or gamma or kappa or lambda or "P.1" or "C.37")	121
#4	"B.1.1.7" or "B.1.351" or "B.1.617.1" or "B.1.617.2"	8
#5	wuhan and (pandemic or corona* or pneumonia)	284
#6	#1 or #2 or #3 or #4 or #5	6175
#7	MeSH descriptor: [Pregnancy Complications] explode all trees	12263
#8	MeSH descriptor: [Pregnancy Outcome] explode all trees	3750
#9	MeSH descriptor: [Obstetric Labor Complications] explode all trees	4070
#10	MeSH descriptor: [Obstetric Labor, Premature] explode all trees	2259
#11	MeSH descriptor: [Fetal Death] explode all trees	367
#12	("adverse birth outcome" or stillbirth* or "low birthweight" or "low birth weigh" or "small for gestational age" or SGA or macrosomia or "neonatal death" or "congenital anomalities"):ti	771
#13	("spontaneous abortion"):ti	139
#14	((labor or labour or deliver* or childbirth* or birth*) Near 3 (prematur* or pre-mature* or preterm or pre-term or early or difficult*)):ti,ab	1773
#15	#7 or #8 or #9 or #10 or #11 #12 or #13 or #14	15308
#16	#6 and #15	16
#17	MeSH descriptor: [Mental Health] explode all trees	1654
#18	MeSH descriptor: [Mental Disorders] explode all trees	75966
#19	MeSH descriptor: [Psychology] explode all trees	1090
#20	MeSH descriptor: [Anxiety] explode all trees	8306
#21	MeSH descriptor: [Depression] explode all trees	12932
#22	((mental* near 3 (ill* or well* or disorder* or disease* or health* or unwell*))):ti	31
#23	((psychiatric* near 3 (ill* or well* or disorder* or disease* or health* or unwell*))):ti	4
#24	(("dysthymic disorder" or "use disorder" or "psychotropic drug" or "brain disease" or "neurotransmitter agent" or cognitive or "social problems" or "alcohol abuse" or "drug abuse")):ti	29197

#25	("seasonal affective disorder" or "internalising symptoms" or "internalizing symptoms" or "common mental disorders"):ti	354
#26	#17 or #18 or #19 or #20 or #21 or #22 or #23 or #24 or #25	108957
#27	#16 and #26	5

PROSPERO Searched July 27, 2021

Line	Search for	Hits
#1	(((coronavirus or corona-virus) AND (wuhan or beijing or shanghai or Italy or South-Korea or korea or China or Chinese or 2019-nCoV or nCoV or COVID-19 or Covid19 or SARS-CoV* or SARSCov2 or ncov)) OR (pneumonia AND Wuhan) or "COVID-19" or "2019-nCoV" or "SARS-CoV" or SARSCOV2 or 2019-nCov or "2019 coronavirus" or "2019 corona virus" or covid19 or ncov OR "novel corona virus" or "new corona virus" or "nouveau corona virus" or "2019 corona virus" OR "novel coronavirus" or "new coronavirus" or "nouveau coronavirus" or "2019 coronavirus") NOT Animal:DB	4745
#2	"birth outcome*" OR "birth complication*" OR stillbirth* OR "fetal death* OR "low birthweight*" OR "low birth weigh" OR "small for gestational age" OR sga OR macrosomia OR "neonatal death" OR "congenital anomal*" OR "pregnancy outcome*" OR "obstetrical outcome*" OR "obstetrical complication*" OR "labor complication*" OR "spontaneous abortion*" NOT Animal:DB	3042
#3	"difficult labo*" or "premature labo*" or "preterm labo*" or "early labo*" NOT Animal:DB 247	
#4	"difficult birth*" or "premature birth*" or "preterm birth*" or "early birth*" or "immature birth*" NOT Animal:DB	1286
#5	"difficult childbirth*" or "premature childbirth*" or "preterm childbirth*" NOT Animal:DB	0
#6	"obstetrical complication*" or "fetus death*" or stillbirth* NOT Animal:DB	876
#7	"mental ill*" or "mental* well*" or "mental disease*" or "mental* disorder*" or "mental* unwell*" NOT Animal:DB	5075
#8	"psychiatric* disease*" or "psychiatric* ill*" or "psychiatric* disorder*" or "psychiartric* health*" NOT Animal:DB	1658
#9	anxiety OR depression OR depressive OR hypervigilance OR agoraphobia OR neuroses OR neurosis OR neurotic OR paranoi* OR catastrophiz* OR catastorphis*or AND obsessive-compulsive OR panic OR phobia OR stress OR wellness OR "seasonal affective disorder*" NOT Animal:DB	9190
#10	"use disorder" OR "psychotropic drug*" OR "brain disease*" OR "neurotransmitter agent*" OR cognitive OR "social problems" OR "alcohol abuse*" OR "drug abuse*" NOT Animal:DB	11626
#11	#3 OR #4 OR #5 OR #6 OR #2	3557
#12	#7 OR #8 OR #9 OR #10	20246
#13	#1 AND #11 AND #12	13
